# La Medicina Familiar en Iberoamérica: voces, sentidos e identidad profesional desde un estudio fenomenológico

**DOI:** 10.1016/j.aprim.2025.103293

**Published:** 2025-05-15

**Authors:** Silvana María Trujillo Cárdenas, Mónica Nieves Martínez Ríos, Cristian Yecid Goyeneche Casanova, Karen Muñoz-Chamorro, Karla Jimena Ortiz Lozano

**Affiliations:** Fundación Universitaria de Ciencias de la Salud, Bogotá, Colombia

**Keywords:** Medicina Familiar, Atención Primaria de Salud, Identidad profesional, Rol profesional, Historia de la Medicina, Family Practice, Primary Health Care, Professional identity, Professional role, History of Medicine

## Abstract

**Introducción:**

La Medicina Familiar ha sido esencial para ofrecer atención continua y holística a individuos y familias, abordando tanto los aspectos físicos como emocionales de la salud a lo largo del curso de vida.

**Objetivo:**

Analizar la evolución de la Medicina Familiar en Iberoamérica y su relación con la identidad profesional del médico especialista y del médico en formación.

**Diseño:**

Estudio cualitativo con enfoque fenomenológico y perspectiva interpretativa-explicativa.

**Lugar:**

Iberoamérica: España, México, Costa Rica, Colombia, Cuba, Perú, Chile, Argentina, Uruguay, Brasil y Paraguay.

**Participantes:**

Doce referentes iberoamericanos en Medicina Familiar con formación de posgrado y amplia experiencia profesional. La selección fue realizada mediante muestreo opinático hasta alcanzar la saturación de la información.

**Métodos:**

Se aplicaron entrevistas semiestructuradas grabadas en audio y video, complementadas con revisión de fuentes bibliográficas. El análisis de los datos se realizó con el software ATLAS.ti versión 24, a través de una codificación sistemática y triangulación de información para contrastar perspectivas y garantizar la validez de los hallazgos.

**Resultados:**

Los entrevistados destacaron que la Medicina Familiar surgió en los años 50 como respuesta a la necesidad de una especialidad generalista con enfoque holístico y preventivo. Se identificaron como elementos clave la atención centrada en la persona y la familia, la continuidad del cuidado, la integralidad, la formación continua y la colaboración con otras disciplinas. La identidad profesional del médico familiar se construye desde la residencia y se fortalece a través de la práctica y la docencia. Además, emergieron más de 100 categorías de análisis útiles para investigaciones futuras.

**Conclusiones:**

La Medicina Familiar representa un pilar esencial de la Atención Primaria en Iberoamérica. A pesar de los desafíos generados por reformas del sistema y políticas de salud, se han consolidado avances significativos en formación, equidad en la atención y consolidación de la identidad profesional. El futuro de la especialidad se vislumbra marcado por la integración digital, la colaboración interprofesional y la humanización del cuidado, preparando médicos adaptables y comprometidos con la salud comunitaria.

## Puntos clave

– Evolución histórica de la Medicina Familiar desde los años 50 hasta la actualidad en Iberoamérica.
– La Medicina Familiar fortalece la Atención Primaria mediante un enfoque integral, continuo y centrado en la persona y su familia.
– La identidad profesional del médico familiar se construye desde la formación y se consolida con la práctica colaborativa e interprofesional.
– El futuro de la especialidad se orienta hacia la integración tecnológica, la humanización del cuidado y el compromiso con la salud comunitaria.

## Abstract (en)

**Introduction:**

Family medicine has been essential in providing continuous and holistic care to individuals and families, addressing both physical and emotional aspects of health throughout the life course.

**Objective:**

To analyze the evolution of family medicine in Ibero-America and its relationship with the professional identity of specialist and trainee physicians.

**Design:**

Qualitative study with a phenomenological approach and an interpretive-explanatory perspective.

**Setting:**

Ibero-America: Spain, Mexico, Costa Rica, Colombia, Cuba, Peru, Chile, Argentina, Uruguay, Brazil, and Paraguay.

**Participants:**

Twelve Ibero-American leaders in family medicine with posgraduate training and extensive professional experience. Participants were selected through purposive sampling until data saturation was reached.

**Methods:**

Semi-structured interviews were conducted and complemented with bibliographic review. Data analysis was performed using ATLAS.ti version 24, applying systematic coding and triangulation to validate the findings.

**Results:**

Interviewees highlighted that family medicine emerged in the 1950s as a response to the need for a generalist, holistic, and preventive specialty. Key elements identified include person- and family-centered care, continuity of care, comprehensiveness, ongoing training, and collaboration with other disciplines. Professional identity is built during residency and strengthened through practice and teaching. Over 100 analytical categories were identified for future research.

**Conclusions:**

Family medicine represents a cornerstone of primary health care in Ibero-America. Despite challenges posed by health system reforms and policies, significant advances have been made in training, equity in care, and consolidation of professional identity. The future of the specialty is marked by digital integration, interprofessional collaboration, and the humanization of care, preparing adaptable physicians committed to community health.

## Introducción

La Medicina Familiar es una especialidad médica que ofrece atención continua e integral a individuos y familias, aborda todas las edades, sexos y sistemas orgánicos^1^. Su historia se remonta a la antigüedad, con enfoques distintos sobre el cuidado del paciente y el concepto de enfermedad. En el siglo XIX, la medicina general era predominante, pero en el siglo XX surgieron las especialidades médicas, lo que llevó a la creación de la Medicina Familiar, en respuesta a la fragmentación de la atención médica^1^. En diferentes países, como Estados Unidos, Canadá, México, Brasil, Cuba, España y Colombia, se han desarrollado programas de formación en Medicina Familiar y se han implementado políticas para fortalecer esta especialidad, reconociendo su importancia en la Atención Primaria de salud^1^, ^2^. Su desarrollo ha estado vinculado a luchas por la equidad y el derecho a la salud, aunque persisten desafíos relacionados con su implementación, el reconocimiento profesional y las condiciones de formación y trabajo.

Este artículo se acerca a la evolución histórica de la Medicina Familiar en la región y su relación con la identidad profesional de quienes la ejercen, mediante revisión bibliográfica y entrevistas a referentes del campo. El objetivo es difundir el papel del especialista en Medicina Familiar dentro del sistema de salud y promover su reconocimiento y comprensión tanto entre profesionales de la salud como entre la población en general. Se realizaron entrevistas semiestructuradas a referentes iberoamericanos de la Medicina Familiar para relatar la historia de la Medicina Familiar en Iberoamérica en los últimos años, destacando su importancia en la Atención Primaria de salud y su impacto en la identidad de los especialistas y médicos en formación^3^.

## Método

Se realizó un estudio de enfoque cualitativo, con perspectiva fenomenológica e interpretativa-explicativa, centrado en la percepción de referentes en Medicina Familiar. El diseño muestral se obtuvo hasta obtener la saturación fenomenológica o temática, con la aplicación de entrevistas semiestructuradas, dirigidas a expertos con formación de posgrado y amplia trayectoria en el campo.

Se llevaron a cabo entrevistas semiestructuradas con 12 referentes iberoamericanos en Medicina Familiar provenientes de países como Costa Rica, Colombia, Perú, Chile, Uruguay, Brasil, Cuba, España, México y Paraguay. La selección se basó en la visibilidad académica de los participantes, la muestra no tuvo carácter representativo, sino teórico-intencional. La muestra fue intencional a conveniencia y se definió mediante criterios de inclusión orientados a garantizar la experiencia y pertinencia de los entrevistados. Se seleccionaron referentes nacionales e internacionales en Medicina Familiar que cumplieran con los siguientes requisitos: contar con formación de posgrado en Medicina Familiar, trayectoria académica o profesional reconocida en su país o en la región y presencia en bases como ORCID o Google Scholar. Todos los participantes firmaron un consentimiento informado previo a la entrevista.

Las entrevistas se realizaron por Google Meet y se registraron en audio y vídeo, complementándose con fuentes bibliográficas pertinentes. La triangulación metodológica permitió contrastar y validar los conceptos emergentes desde distintas perspectivas y contextos nacionales.

Se tomaron medidas para minimizar sesgos. Para el sesgo de selección, se eligieron referentes que cumplían con los criterios de inclusión y aceptaron voluntariamente participar. Para reducir el sesgo de información, se utilizó un guion semiestructurado enfocado en los objetivos del estudio, se garantizó anonimato y se promovió un ambiente de confianza. Adicionalmente, algunos hallazgos fueron validados (se retornó) con los entrevistados para asegurar la fidelidad en la interpretación de sus perspectivas.

Las investigadoras responsables del trabajo de campo contaban con formación en investigación cualitativa, lo que contribuyó a minimizar errores en la formulación de preguntas, en el registro de información y en la interpretación de las respuestas. Las entrevistas se realizaron siguiendo una guía previamente elaborada y validada por el equipo investigador y fueron grabadas, transcritas y analizadas de manera sistemática.

Se trabajó con categorías preestablecidas (historia de la Medicina Familiar, identidad profesional, formación profesional, práctica laboral, visión futura e impacto en el modelo de salud de la Medicina Familiar). El análisis se realizó con ATLAS.ti versión 24, que permitió una codificación sistemática, incluyendo el uso de inteligencia artificial, lo que generó más de 100 categorías emergentes. Para este estudio, se utilizaron las categorías definidas por el equipo investigador.

**Figura 1:**
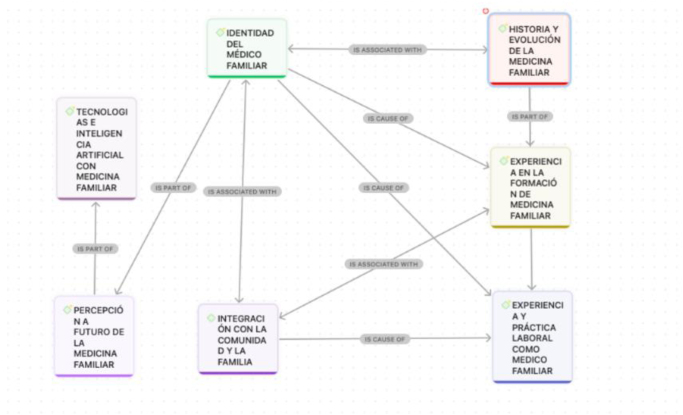
Red de conceptos generados por ATLAS.ti.

La triangulación de datos permitió un análisis narrativo de contenido por parte del equipo investigador, asegurando rigor y validez. Este enfoque facilitó la construcción de una teoría fundamentada en las percepciones de los participantes, ofreciendo una visión profunda sobre la Medicina Familiar y la identidad profesional. El software generó una red de códigos que organizó visualmente los conceptos extraídos **(**fig. 1).

## Resultados y discusión

A continuación, se presentan los hallazgos organizados por categorías.

### Categoría: Historia de la Medicina Familiar

Los entrevistados coinciden en ubicar el origen de la Medicina Familiar en Iberoamérica entre las décadas de 1950 y 1960, como respuesta a problemáticas sanitarias y sociales emergentes. Relatan que en diferentes países comenzó a consolidarse una atención médica centrada en la comunidad, con antecedentes en la figura del médico generalista o itinerante, enfatizan que uno de los elementos distintivos de la Medicina Familiar es su enfoque centrado en la persona, más allá del diagnóstico o la enfermedad. Destacan también la integración del modelo biopsicosocial como fundamento de su práctica profesional.

*«La atención centrada en la persona es lo principal. La otra es que nosotros tenemos un enfoque biopsicosocial que no tienen otras especialidades, y otra que nos hace diferentes es la posibilidad de seguir en esa longitudinalidad del proceso de vida de las personas a lo largo del ciclo vital».* (Entrevistado 3).

*«La Medicina Familiar es una especialidad médica que tiene un amplio bagaje de aplicaciones y que principalmente se basa en una atención integrada y basada en la persona. Tiene un enfoque holístico biopsicosocial que pretende buscar bienestar a la persona en todo su ámbito».* (Entrevistado 5).

Otro de los aspectos mencionados con fuerza es la longitudinalidad, es decir, la continuidad en la atención del mismo paciente o familia a lo largo del tiempo, lo cual refuerza la identidad profesional del médico familiar.

*«... tener el mismo médico de familia durante más de 15 años tiene una disminución de su mortalidad entre un 25 y un 30%. Es decir, esto es fundamental para tener identidad como médico de familia. Que soy una médico de personas, que estoy centrada en la persona, en su familia y en su comunidad».* (Entrevistado 6).

Los entrevistados ubican el origen de la Medicina Familiar en Iberoamérica como respuesta a desafíos sanitarios y sociales. Rescatan antecedentes en el médico generalista o de cabecera, que brinda atención integral y cercana a las familias.

*«En México el origen de la Medicina Familiar data de 1953 [...] donde surge el problema que hace requerir esta especialidad».* (Entrevistado 1).

*«Realmente la Medicina Familiar se originó de aquellos primeros médicos de cabecera [...] el médico era quien se acercaba a las familias para solucionar una situación».* (Entrevistado 10).

A partir de entonces, se consolidó como especialidad centrada en la persona y su contexto, con enfoque preventivo, presencia en todos los niveles del sistema y continuidad en el tiempo.

*«Se concibe como especialidad médica más o menos en los años 1969 con una mirada más amplia del sistema de salud»*. (Entrevistado 4).

Este enfoque integral es destacado como rasgo distintivo: «Tenemos un enfoque biopsicosocial que no tienen otras especialidades... y la posibilidad de seguir en esa longitudinalidad del proceso de vida». (Entrevistado 3).

*«La Medicina Familiar principalmente se basa en una atención integrada y basada en la persona».* (Entrevistado 5).

Los testimonios muestran que la Medicina Familiar surgió vinculada a movimientos de reforma y a una visión comunitaria de la salud. Su historia se construye desde una identidad profesional centrada en la persona, la familia y la comunidad, con un compromiso sostenido con la continuidad del cuidado y la transformación de los sistemas de salud.

### Categoría: Identidad del médico familiar

Los entrevistados coinciden en que la identidad del médico familiar se define por su enfoque integral, la continuidad del cuidado y la cercanía con el paciente, su familia y su entorno.

*«Un factor diferenciador es la integralidad debemos ser de manera continua el médico del paciente».* (Entrevistado 2).

*«Atiende a diferentes grupos de edad... con la posibilidad de tener la continuidad de la atención».* (Entrevistado 10).

También destacan su rol en promoción y prevención de la salud.

*«Es un imperativo ético ver qué sucede en el entorno familiar y generar estrategias preventivas y promocionales [...] es parte del quehacer diario».* (Entrevistado 4).

Este compromiso se refuerza con la presencia territorial: *«El médico familiar debe residir en el mismo hábitat de sus pacientes [...] mientras no tengamos la Atención Primaria como centro del sistema, no lo vamos a poder desarrollar».* (Entrevistado 9).

Otro rasgo de identidad es la capacidad resolutiva: *«Eso es lo que nos hace diferentes: la capacidad de resolver problemas».* (Entrevistado 6). *«Hace énfasis en el manejo integral y humanístico de las situaciones de salud del paciente, la familia y la comunidad».* (Entrevistado 7).

La formación en residencia es clave para consolidar esta identidad, pese a las barreras simbólicas: *«La identidad se va forjando desde la residencia [...] con el tiempo se dan cuenta cómo aumentan sus competencias».* (Entrevistado 10).

Los relatos muestran que la identidad del médico familiar se construye en tensión entre el ideal de una atención integral, resolutiva y centrada en la persona, y las limitaciones estructurales del sistema. La precariedad laboral, la falta de reconocimiento y la escasa visibilidad afectan el ejercicio profesional, a pesar del amplio campo de acción que abarca también gestión, docencia e investigación. Esta visión es coherente con las recomendaciones internacionales que impulsan un modelo centrado en valores éticos, arraigo territorial y uso crítico de nuevas tecnologías. La inteligencia artificial, por ejemplo, es reconocida como herramienta útil, pero no sustituta del vínculo humano que define esta especialidad.

### Categoría: Experiencia en la formación en Medicina Familiar

La formación en Medicina Familiar es percibida como un proceso exigente que articula conocimientos clínicos, práctica comunitaria y formación en valores.

*«Debe ser 50% Medicina Familiar y temas médicos, y el otro 50% práctica en comunidad».* (Entrevistado 1).

Desde etapas tempranas, se promueve un enfoque longitudinal del cuidado: *«La formación comienza desde el primer año en un consultorio médico [...] desde el neonato usted empieza a seguirlo».* (Entrevistado 9).

La presencia del médico familiar en distintos niveles del sistema permite visibilizar su aporte y construir vínculos interprofesionales.

*«Ha permitido que los residentes de las demás especialidades [...] entiendan la importancia de esta especialidad».* (Entrevistado 3).

También se destaca la capacidad resolutiva como eje del perfil formativo: *«Podemos resolver hasta el 95% de las consultas [...] ese paciente ya va orientado».* (Entrevistado 3).

El trabajo en red se presenta como otro pilar esencial: *«No nos podemos construir como especialistas solos, sino con otras disciplinas y especialidades».* (Entrevistado 4). En este proceso, la figura del tutor adquiere un rol central en la construcción identitaria: *«Debe tener tutores que realmente sepan y vivan qué es la Medicina Familiar».* (Entrevistado 3). *«Cuando tienen tutores de la especialidad ven cómo es la utilidad real [...] se crea un carisma particular».* (Entrevistado 10).

Las entrevistas reflejan una visión compartida sobre la Medicina Familiar como práctica integral, humana y con fuerte anclaje territorial. Este enfoque formativo dialoga con el surgimiento histórico de la especialidad como respuesta a la fragmentación del sistema, recuperando el sentido social y comunitario de la medicina. La formación se estructura en torno a competencias clínicas, sensibilidad social y capacidad de liderazgo, en línea con los marcos de referencia internacionales.

No obstante, persisten desafíos como la estandarización curricular, la incorporación de contenidos clave y la consolidación de programas más equitativos. Se valora la inclusión de actividades en comunidad, la figura del tutor especializado y el desarrollo de habilidades para la atención integral y la resolución de problemas. Estos elementos son consistentes con el perfil profesional propuesto por organismos como la Organización Panamericana de la Salud (OPS) y la Federación Iberoamericana de Medicina Familiar (FIMF). En síntesis, la formación en Medicina Familiar se configura como un espacio de construcción identitaria y compromiso ético, atravesado por tensiones estructurales, pero con alto potencial transformador en los sistemas de salud.

### Categoría: Experiencia y práctica laboral como médico familiar

El ejercicio profesional del médico familiar se caracteriza por su versatilidad y compromiso transversal con el sistema de salud. Desde la consulta clínica hasta la docencia y la gestión, estos profesionales asumen múltiples funciones en distintos niveles de atención. Su práctica cotidiana está marcada por un abordaje centrado en la persona, la familia y el entorno:

*«Al atender pacientes enfocado en la familia y no en el individuo, integramos lo que es la Medicina Familiar».* (Entrevistado 1).

Las visitas domiciliarias, la promoción de la salud y la prevención son componentes centrales de su labor:

*«Yo me tomaba casi una hora con ellos en la consulta, y cuando no podía, entonces iba a sus casas».* (Entrevistado 8).

*«Realizamos visitas domiciliarias con el equipo, salimos a mirar cómo son las dinámicas familiares».* (Entrevistado 12).

También se reconoce su rol en la gestión eficiente de recursos y en la construcción de vínculos terapéuticos:

*«Demostré las habilidades para mantener a la población satisfecha, adherente al tratamiento y al médico».* (Entrevistado 7).

Su desempeño se extiende al ámbito intersectorial, la formación académica y la gestión institucional:

*«Trabajo en el hospital, en educación virtual del ministerio, soy docente y hago consultorio».* (Entrevistado 10).

Algunos entrevistados destacaron también su participación en espacios internacionales:

*«Fui elegida como mejor médico de familia del mundo».* (Entrevistado 6). *«Logré ser presidenta de la Confederación Iberoamericana de Medicina Familiar».* (Entrevistado 12).

Todo ello sostenido en valores fundamentales como la humildad, la apertura y el trabajo en equipo:

*«Lo más importante que debe tener un médico familiar es la humildad y permitir la participación de los demás».* (Entrevistado 12).

Las narrativas reflejan una práctica laboral multidimensional, coherente con la literatura que describe a la Medicina Familiar como una especialidad con fuerte anclaje en el primer nivel, liderazgo comunitario y capacidad para articular múltiples funciones^4^, ^5^, ^6^. La integración de actividades clínicas, educativas, de gestión e investigación posiciona al médico familiar como un actor clave en el entramado sanitario. Sin embargo, los relatos también evidencian tensiones estructurales, como la precariedad laboral y la escasa participación en decisiones políticas, desafíos que coinciden con estudios previos^6^, ^7^, ^8^ y que requieren acciones decididas para potenciar su desarrollo.

### Categoría: Percepción a futuro de la Medicina Familiar y su impacto con el modelo de salud

Los entrevistados coinciden en que la Medicina Familiar tiene un papel clave en la sostenibilidad de los sistemas de salud del futuro. Su enfoque integral y centrado en la persona la convierte en una disciplina estratégica para enfrentar los desafíos sanitarios contemporáneos:

*«Veo la Medicina Familiar como la medicina del futuro».* (Entrevistado 3). *«El futuro de la salud está en la cabeza de los médicos familiares».* (Entrevistado 7).

No obstante, su consolidación depende de su inclusión real en los modelos de salud y políticas públicas de cada país. En cuanto al uso de tecnologías emergentes como la inteligencia artificial, si bien se reconoce su utilidad, se advierte sobre sus limitaciones:

*«Son herramientas que no pueden sustituir nunca el contacto».* (Entrevistado 6). *«Tenemos que aprender cómo hacer para que esas tecnologías no nos superen».* (Entrevistado 12).

Las visiones recogidas combinan esperanza y crítica, reafirmando el potencial transformador de la Medicina Familiar sin desconocer las barreras que enfrenta. Este equilibrio entre ideal y realidad se alinea con las recomendaciones de organismos internacionales que promueven sistemas de salud centrados en valores éticos, equidad y tecnologías apropiadas^9^, ^10^, ^11^. La Medicina Familiar, tal como la viven sus protagonistas, representa una apuesta por la humanización, la equidad y la sostenibilidad en salud. Para que este potencial se concrete, es necesario fortalecer su inserción en las políticas públicas, garantizar condiciones laborales dignas y acompañar su evolución con recursos adecuados.

En síntesis, la triangulación entre los discursos recogidos y la bibliografía especializada permite afirmar que la Medicina Familiar en Iberoamérica es vivida como una práctica profundamente humana, con un fuerte sentido social, aunque aún enfrenta barreras estructurales que deben ser abordadas para fortalecer su desarrollo y proyección futura.

## Limitaciones

Este estudio presenta algunas limitaciones que deben tenerse en cuenta al interpretar los resultados. En primer lugar, la inclusión de un solo referente por país constituye una limitación desde el punto de vista investigativo. No obstante, se trata de figuras con activa participación en redes internacionales, cuya experiencia y capacidad de análisis aportan una visión amplia y contextualizada. En todos los casos, se realizó una devolución de la información y de las categorías emergentes a los propios entrevistados, sin que se manifestaran objeciones ni disconformidades, lo que refuerza la validez del análisis interpretativo.

Un aspecto relevante es que, al tratarse de un estudio cualitativo con enfoque fenomenológico, no se buscó establecer relaciones causa-efecto, no se buscó establecer relaciones causa-efecto, sino comprender significados y experiencias desde la perspectiva de los propios referentes académicos y asistenciales. Esto implica que los resultados deben interpretarse en el marco de este enfoque, sin pretensiones de generalización.

Finalmente, el estudio no tiene como objetivo realizar comparaciones detalladas entre los sistemas de salud de los países participantes, ni abordar de manera sistemática los procesos de formación en Medicina Familiar, aunque se mencionan aspectos relacionados con la enseñanza y la docencia. Este enfoque limita el alcance en cuanto a ofrecer una visión exhaustiva o comparativa sobre la situación de la Medicina Familiar en la región.

## Conclusiones

Este estudio cualitativo, de enfoque fenomenológico y con una perspectiva interpretativa-explicativa, permitió explorar las percepciones y experiencias de médicos de familia con una destacada trayectoria académica y asistencial en Iberoamérica. El análisis de sus testimonios reveló los significados que estos referentes atribuyen a su práctica profesional, su identidad, los desafíos que enfrentan y las proyecciones que vislumbran para la especialidad en la región.

A lo largo del artículo, las principales conclusiones se han desarrollado en cada categoría analizada; por lo tanto, esta sección no busca reiterarlas, sino integrarlas. A través de las categorías emergentes, se evidenció el potencial transformador de la Medicina Familiar, especialmente en el fortalecimiento de la Atención Primaria. Sin embargo, también se identificaron barreras estructurales significativas que dificultan su consolidación, tales como la fragmentación de los sistemas de salud, las condiciones laborales precarias, el escaso reconocimiento institucional y la limitada participación en la toma de decisiones. Estas dificultades, aunque no siempre expresadas de forma explícita, fueron subrayadas por las experiencias compartidas por los entrevistados.

Este trabajo no se centró en realizar comparaciones detalladas entre los contextos nacionales ni en abordar de manera exhaustiva los procesos formativos en Medicina Familiar. Tampoco pretende describir en detalle la situación actual de la Medicina Familiar en cada país participante. Aunque se incluyeron algunas referencias a la docencia como parte del quehacer profesional, no se profundizó en los procesos formativos de manera sistemática. Además, se optó por emplear la denominación de *«Medicina Familiar»* en lugar de *«Medicina Familiar y Comunitaria»* debido a las diferencias en la denominación oficial entre países. En algunos contextos, como en España, se utiliza la expresión completa, mientras que en otros se prefiere la forma abreviada. Por razones de coherencia terminológica y comprensión regional, se adoptó el término más comúnmente usado en los países participantes.

No obstante, este trabajo aporta una visión contextualizada y rica sobre cómo se vive y se proyecta esta especialidad en Iberoamérica. En consecuencia, sus hallazgos contribuyen al debate académico, al enriquecimiento de la política pública y al fortalecimiento de la Medicina Familiar como una especialidad esencial para avanzar hacia sistemas de salud más integrales, humanos y sostenibles.

## Declaraciones de ética

Declaramos que nuestro trabajo no ha sido experimentado en animales, no interviene en pacientes humanos, no es un ensayo clínico y todos los datos se recogen en el apartado de resultados y conclusiones.

## Financiación

El presente proyecto fue financiado por los autores.

## Conflicto de intereses

Los autores declaran que no tienen intereses financieros en conflicto ni relaciones personales conocidas que pudieran haber influido en el trabajo presentado en este artículo.
